# Characteristics of carpal tunnel syndrome in patients with cervical radiculopathy: A cross‐sectional study

**DOI:** 10.1002/hsr2.1575

**Published:** 2023-09-20

**Authors:** Alireza Teymouri, Seyede Zahra Emami Razavi, Mohaddeseh Azadvari, Maryam Hosseini

**Affiliations:** ^1^ Department of Physical Medicine and Rehabilitation, Imam Khomeini Hospital Complex Tehran University of Medical Sciences Tehran Iran; ^2^ Department of Physical Medicine and Rehabilitation, Sina Hospital Tehran University of Medical Sciences Tehran Iran

**Keywords:** carpal tunnel syndrome, cervical radiculopathy, double crush syndrome, orthopedics, physical therapy

## Abstract

**Background and Aims:**

Cervical radiculopathy (CR) is a group of signs and symptoms caused by cervical root dysfunction. Patients with this condition may also have carpal tunnel syndrome (CTS), which is caused by compression of the median nerve in the carpal tunnel. This coexistent condition is referred to as double crush syndrome (DCS) which is explained by proximal disruption in axoplasmic flow that may result in nerve dysfunction. Yet, the accuracy of this hypothesis remains controversial.

**Materials and Methods:**

Patients with confirmed CR according to electromyography were included in this retrospective study. However, we omitted patients with overt diabetic neuropathy, prior spinal or hand surgery and/or fractures, and rheumatoid arthritis. Patients underwent electrodiagnostic studies, and the results were used to determine CTS severity. We used Pearson's *χ* ^2^ test to assess the correlation between the severity of CTS and CR. Descriptive analysis was used to report patient characteristics and the prevalence of CTS in CR patients.

**Results:**

We included 291 participants, 59% of whom were women. Although insignificant, DCS was observed more in women (*n* = 110) compared to men (*n* = 71). However, we found that patients with DCS (54.81 ± 13.3) were older than non‐DCS patients (43.71 ± 12.94) which was statistically significant (*p* < 0.05). There was statistically no correlation between the severity of CR and CTS, ipsilaterlly (*p* > 0.05). In total, CTS was observed in 62.2% (*n* = 181) patients.

**Conclusion:**

In summary, we found a relatively high prevalence of DCS in the present study. In patients with and without DCS, gender did not seem to play a role but the growing age seemed to contribute to DCS. The severity of CTS was not related to CR severity at any cervical level, which negates a causal relation between the pre‐existing CR and newly diagnosed CTS.

## 1 INTRODUCTION

Nerve entrapment syndromes are peripheral neuropathies with structural changes in nerves due to microvascular disruption. Carpal tunnel syndrome or CTS is a common diagnosis that belongs to this category of diseases and must be differentiated from cervical radiculopathy or CR.^1^ CR is mostly due to foraminal narrowing of the spinal nerve (70%–75%), and less frequently, a herniated nucleus pulposus is responsible for the presenting symptoms (20%–25%).^2^ This includes pain, weakness (myotomal distribution), paresthesia, and sensory deficit (along the corresponding dermatome).^3^ Main differential diagnoses of CR include shoulder pathology, and other nerve entrapment syndromes (e.g., CTS or ulnar nerve entrapment).^4^

CTS is the most common nerve entrapment syndrome and it has an incidence of 1–3 persons per 1000 per year.^5^ Patients with CTS, initially complain of numbness and/or unpleasant sensation in fingers. Some of them experience pain and paresthesia in the forearm or shoulder.^6^ The diagnosis is based on characteristic symptoms and physical examination. Electromyography and/or nerve conduction velocity or EMG‐NCV is used to confirm the diagnosis and exclude other conditions like polyneuropathy and radiculopathy as well.^7^ Similar to CR, treatment for CTS includes operative (nerve decompression) and nonoperative methods such as neutral‐angle wrist splinting and steroid injection.^8^

Double crush syndrome (DCS) first was proposed by Upton et al. in 1973. They studied 115 patients with CTS, of which 81 (70%) had concomitant CR. This hypothesis stated that the proximal disruption of the axoplasmic flow might result in multiple injured sites alongside the peripheral nerve.^9^ This phenomenon may also be due to dorsal root ganglia inflammation, ion channel dysregulation, and neuroma in continuity.^10^ In addition, there are several animal studies that support DCS.^11^, ^12^ However, the accuracy of DCS has been questioned multiple times and remains controversial.^13^, ^14^, ^15^, ^16^, ^17^

C6 and C7 nerve roots typically provide sensory innervation to digits 1–3 (the thumb, index, and middle finger), which are the same fingers affected in CTS, whereas C8 supplies digits 4 and 5. Thus, theoretically an isolated sensory deficit in digits 4 and 5 rules out the diagnosis of CTS. Accordingly, impairment in C6 and C7 nerve roots may result in overlapping symptoms and signs in these conditions and lead to misdiagnosis. CR and CTS require distinct treatment approaches at different sites along the upper extremities. In addition, there are many patients who are simultaneously affected by both conditions. These underscore the need to evaluate these patients with more scrutiny to differentiate between CR and CTS. In the present study, we aim to estimate the prevalence of CTS amongst patients with CR and explore the possibility of a positive correlation between the severity of CTS and CR.

## 2 METHODS

The current study is designed as a retrospective cross‐sectional study. We enrolled patients with suspected CR who presented to our clinic between 2017 and 2020. The eligible patients for inclusion in the study were diagnosed with CR which was confirmed with EMG‐NCV.^18^ Patients with overt diabetic neuropathy, prior spinal or hand surgery and/or fractures, and rheumatoid arthritis were not included. We used the following formula for sample size calculation: *n* = *z* ^2^·*p*·*q*/*d* ^2^, where *z* = level of significance at 95% confidence interval (CI) (=1.96), *p* = proportion of patients who have coexisting CTS and CR in the study population (0.24), *q* = 1 – *p* (0.76), *d* = usually a constant set at 0.05. This formula yields 280 which is the minimum sample size for this study.

Patient history acquisition and physical examination (e.g., Tinel and Phalen tests) were carried out in all patients. Despite the clinical picture, all patients were assessed with electrodiagnostic studies or EDX, and an available EDX device (Medelec Premiere Plus) was used to record the results. The temperature of the hand was maintained at or above 32°C. Median Nerve conduction studies were done to assess the initial latency and baseline‐to‐peak amplitude. For this purpose, the abductor pollicis brevis muscle and third finger were used to record the motor and sensory response, respectively. The severity of CR was determined by assessing the reduction in amplitude and clinical manifestations. Furthermore, we categorized the CTS into mild, moderate, and severe based on EMG‐NCV. Patients with CTS which was confirmed with most sensitive tests had mild disease. Moderate severity had normal terminal motor latency with slow sensory NCV, whereas, in the severe form of the disease, there was an overt increase in distal motor latency to abductor pollicis brevis.^19^

The analysis in this study was done via SPSS Statistics (version 19) and results were reported with a CI of 95%. A *p* value less than 0.05 was considered significant. Demographic data including age and sex were extracted from patients' records. The Kolmogorov–Smirnov test was run to assess the normality of distribution in quantitative data. Pearson's *χ* ^2^ test and paired *T* test were carried out to assess the correlation between the different variables. We acquired informed consent from each patient, and the data was anonymously codified. This study was approved by the Ethical Committee of the Tehran University of Medical Sciences and it is registered under IR.TUMS.IKHC.REC.1398.245.

## 3 RESULTS

### 3.1 Patient characteristics

The participants in this study were 291 patients with definite CR who presented to our physical medicine and rehabilitation clinic. Of these, 120 (41.2%) were men and 171 (58.8%) were women. The mean age was 50.6 ± 14.2 years. Bilateral, right, and left CR was seen in 196, 248, and 239 patients, respectively. A summary of CR and CTS characteristics is provided in Table 1. Other radiculopathies including lumbosacral, ulnar, and radial radiculopathies accounted for 20.3% (59), 8.3%^20^, and 0.7%,^2^ respectively. Of the patients, 4.5%^13^ had diabetes mellitus on oral medication with no overt diabetic nephropathy.

**Table 1:** Characteristics of CR and CTS in the study population.

CR and CTS	No (%)	Yes (%)	Mild (%)	Moderate (%)	Severe (%)
Right C6	201 (69.1)	90 (30.9)	66 (22.7)	19 (6.5)	5 (1.7)
Right C7	130 (44.7)	161 (55.3)	128 (44)	31 (10.7)	2 (0.7)
Right C8	100 (34.4)	191 (65.6)	153 (52.6)	34 (11.7)	4 (1.4)
Left C6	196 (67.4)	95 (32.6)	76 (26.1)	17 (5.8)	2 (0.7)
Left C7	133 (45.7)	158 (54.3)	129 (44.3)	28 (9.6)	1 (0.3)
Left C6	111 (38.1)	180 (61.9)	157 (50.5)	32 (11)	1 (0.3)
Right CTS	118 (40.5)	173 (59.5)	49 (16.8)	101 (34.7)	23 (7.9)
Left CTS	123 (42.3)	168 (57.7)	55 (18.9)	89 (30.6)	24 (8.2)

Abbreviations: CR, cervical radiculopathy; CTS, carpal tunnel syndrome.

### 3.2 Descriptive data

We observed that C8 was affected more compared to C6 or C7 in CR patients. For instance, right‐sided CR at C6 and C8 levels accounted for 30.9% (90) and 65.6% (191), respectively. In terms of severity, the majority of participants had mild or moderate disease. A total of 187 (64.3%) patients had CTS, 33 of them were unilateral and 154 were bilateral. Similar to CR, the prevalence of mild or moderate CTS was higher in comparison to its severe form.

### 3.3 Main results

As for the primary endpoint of this study, we calculated the prevalence of ipsilateral CTS in CR patients, and 181 (62.2%) of them had DCS. So, 6 patients had CTS contralateral to CR involvement. CTS in the right hand, left hand, and bilaterally was seen in 156 (53.6%), 141 (48.5%), and 116 (39.9%) patients, respectively. The mean age of the patients with and without DCS was 54.81 ± 13.3 and 43.71 ± 12.94, respectively, which were significantly different (*p* < 0.001). A higher number of DCS patients were women (110 vs. 71), but there was no statistical difference (*p* = 0.37). Lastly, we combined the moderate and severe subgroups for CTS and CR because of the limited number of patients in each category and analyzed the correlation between CR and CTS severity. There was no significant association between the severity of CTS and CR at each nerve root level (*p* > 0.05). However, this insignificance was less at the C6 nerve root level (*p* = 0.10). These findings are summarized in Table 2.

**Table 2:** Results of Pearson's *χ* ^2^ test regarding the severity of CTS and CR at each nerve root level.

CR	Right CTS	
	Mild	Moderate to severe
Right C6 CR		
Mild	14	27
Moderate to severe	2	14
*p* value	0.10	
Right C7 CR		
Mild	22	61
Moderate to severe	4	14
*p* value	0.71	
Right C8 CR		
Mild	26	67
Moderate to severe	4	18
*p* value	0.35	

	**Left CTS**	
	Mild	Moderate to severe
Left C6 CR		
Mild	13	29
Moderate to severe	2	11
*p* value	0.27	
Left C7 CR		
Mild	21	59
Moderate to severe	4	12
*p* value	0.92	
Left C8 CR		
Mild	32	54
Moderate to severe	5	13
*p* value	0.45	

Abbreviations: CR, cervical radiculopathy; CTS, carpal tunnel syndrome.

## 4 DISCUSSION

Our study reported a relatively high prevalence of 62.2%, whereas in most studies, the frequency of DCS is low and ranges from 5% to 50%.^17^, ^21^, ^22^ This might be due to the fact that most patients were referred to our clinic from different provinces. Consistent with our findings, the increasing age was associated with DCS presence in most studies.^17^, ^22^, ^23^, ^24^ In our study, DCS was found at 10.6%, 17.5%, and 23% at C6, C7, and C8 levels, respectively. This higher frequency at the C8 level was congruent with Richardson's study (29.1%) and in contrast with Kwon's (3.9%).^13^, ^21^ In a similar study by Kwon et al. with a quantitative approach, 277 patients with CR were evaluated of which 39 (14%) had coexistent CTS. The frequencies of CTS were not statistically different according to nerve root level. They compared the latency and amplitude of motor and sensory responses between proximal nerve roots (C6 and C7) and C8 and found no significant difference. Theoretically, an association between CR and CTS is expected at proximal nerve roots (C6 and C7), but we found no correlation between the severity of CR and CTS at any nerve root level. This was in keeping with quantitative findings based on EDX in other studies as well.^16^, ^20^, ^21^

We can attribute the development of DCS to the fact that CR and CTS may share some risk factors such as diabetes, medications, and some endocrinopathies.^25^ Determining the order in which the DCS is treated is based on the extent of compression and the symptoms at each affected site. If a diagnosis of DCS is made, the surgeon will begin with standard nonsurgical management for each level of suspected nerve compression. Cervical nerve root compression can be treated initially with oral steroids, and avoiding movements that aggravate the condition. For distal lesions, common palliative treatments include steroid injections, splinting, and nonsteroidal anti‐inflammatory drugs. If a patient with DCS does not experience clinical improvement with a carpal tunnel injection, this may indicate that they are unlikely to benefit from carpal tunnel release surgery alone.^26^ According to Osterman's research, 33% of patients with DCS considered carpal tunnel release a failure, compared to only 7% of those with CTS alone.^27^

This study had several limitations. First of all, it was a retrospective cross‐sectional study that in comparison to prospective studies, has a lower ability to draw associations and causal relationships. It was conducted as a single‐center study and the sample size was relatively small. In addition, we did not conduct a quantitative analysis based on latency and amplitude and did not design a regression model adjusting for potentially confounding variables. We recommend multiple‐center prospective studies with quantitative data to clarify the correlation between the two conditions. Additionally, to the best of our knowledge, the pathologic processes that lead to DCS are still unknown and there is no existent standardized guideline for its treatment. Thus, we need translational research and protocol development for treating DCS.

## 5 CONCLUSION

In summary, the prevalence of DCS was relatively high in our study and involved the majority of patients. Increasing age was correlated with the development of DCS, and we found that women are more affected by DCS than men. CTS severity and CR severity were not correlated at the C6, C7, and C8 nerve roots. Thus, we could not demonstrate a causal relation between CR and CTS. Nevertheless, each of these conditions must be evaluated and treated with caution due to their high coexistence.

## AUTHOR CONTRIBUTIONS

**Alireza Teymouri**: Data curation; formal analysis; writing—original draft; writing—review and editing. **Seyede Zahra Emami Razavi**: Conceptualization; data curation; investigation; methodology; validation; writing—review and editing. **Mohaddeseh Azadvari**: Data curation; investigation; project administration; writing—review and editing. **Maryam Hosseini**: Investigation; resources; supervision; validation.

## CONFLICT OF INTEREST STATEMENT

The authors declare they have no conflict of interest.

## TRANSPARENCY STATEMENT

The lead author Seyede Zahra Emami Razavi affirms that this manuscript is an honest, accurate, and transparent account of the study being reported; that no important aspects of the study have been omitted; and that any discrepancies from the study as planned (and, if relevant, registered) have been explained.

## Data Availability

The data that support the findings of this study are available from the corresponding author upon reasonable request.
